# Interleukin-19 Acts as a Negative Autocrine Regulator of Activated Microglia

**DOI:** 10.1371/journal.pone.0118640

**Published:** 2015-03-20

**Authors:** Hiroshi Horiuchi, Bijay Parajuli, Yue Wang, Yasu-Taka Azuma, Tetsuya Mizuno, Hideyuki Takeuchi, Akio Suzumura

**Affiliations:** 1 Department of Neuroimmunology, Research Institute of Environmental Medicine, Nagoya University, Furo-cho, Chikusa-ku, Nagoya, 464–8601, Japan; 2 Laboratory of Veterinary Pharmacology, Division of Veterinary Science, Osaka Prefecture University Graduate School of Life and Environmental Science, Izumisano, Osaka, 598–8531, Japan; Indiana School of Medicine, UNITED STATES

## Abstract

Activated microglia can exert either neurotoxic or neuroprotective effects, and they play pivotal roles in the pathogenesis and progression of various neurological diseases. In this study, we used cDNA microarrays to show that interleukin-19 (IL-19), an IL-10 family cytokine, is markedly upregulated in activated microglia. Furthermore, we found that microglia are the only cells in the nervous system that express the IL-19 receptor, a heterodimer of the IL-20Rα and IL-20Rβ subunits. IL-19 deficiency increased the production of such pro-inflammatory cytokines as IL-6 and tumor necrosis factor-α in activated microglia, and IL-19 treatment suppressed this effect. Moreover, in a mouse model of Alzheimer’s disease, we observed upregulation of IL-19 in affected areas in association with disease progression. Our findings demonstrate that IL-19 is an anti-inflammatory cytokine, produced by activated microglia, that acts negatively on microglia in an autocrine manner. Thus, microglia may self-limit their inflammatory response by producing the negative regulator IL-19.

## Introduction

Microglia are the macrophage-like resident immune cells that contribute to the maintenance of homeostasis in the central nervous system (CNS). Their functions include antigen presentation to initiate the immunological reaction, direct attack against non-self antigens, debris clearance, and support of neuronal circuit development [1–5]. Abnormal activation of microglia often damages the CNS, and microglial activation is a characteristic pathological hallmark of various neurological disorders, including neuroinflammatory and neurodegenerative diseases [6–12]. Activated microglia can exert either neurotoxic or neuroprotective effects, depending on their environmental conditions and the type and extent of stimulation [13–16], and can release both pro-inflammatory cytokines (e.g. interleukin-1β [IL-1β], IL-6, tumor necrosis factor-α [TNF-α]) and anti-inflammatory cytokines (e.g. IL-10, transforming growth factor-β). Pro-inflammatory cytokines exacerbate neuroinflammation by fueling microglial activation, whereas anti-inflammatory cytokines oppose those effects by suppressing microglial production of pro-inflammatory cytokines [17], suggesting that microglial activation is self-limiting.

IL-19 is a member of the IL-10 family cytokines including IL-20, IL-22, IL-24, IL-26, IL-28A, IL-28B, and IL-29 [18]. At the primary sequence level, IL-19 is highly homologous to IL-20 and IL-24, and shares a receptor formed by heterodimerization of the IL-20Rα and IL-20Rβ subunits [19–21]. Previous studies showed that IL-19 contributes to the pathogenesis of autoimmune diseases such as psoriasis, asthma, and rheumatoid arthritis by upregulation of pro-inflammatory cytokines, including IL-6 and TNF-α [22–24]. However, the source and biological functions of IL-19 in the CNS have yet to be elucidated.

In this study, we found that microglia are the predominant producers of IL-19 in the CNS, as well as the only CNS cells to express functional IL-19 receptors. Furthermore, IL-19 suppresses microglial production of pro-inflammatory cytokines. Our findings suggest that microglia self-limit their participation in neuroinflammation and neurodegeneration in various neurological diseases by producing a negative regulator, IL-19.

## Methods

### Reagents

Lipopolysaccharide (LPS) was purchased from Sigma-Aldrich (St. Louis, MO, USA). Recombinant mouse IL-19 protein was purchased from R&D Systems (Minneapolis, MN, USA).

### Animals

All animal experiments were conducted under protocols approved by the Animal Experiment Committee of Nagoya University (approved numbers: 14017, 14018, and 14020). C57BL/6 (B6) mice were purchased from Japan SLC (Hamamatsu, Japan). IL-19^-/-^ mice (B6 background) [25,26] were obtained from Regeneron Pharmaceuticals, Inc. (Tarrytown, NY, USA). Transgenic mice expressing variants of human amyloid precursor protein (APP) with the K595N and M596L mutations and presenilin 1 (PS1) with the A264E mutation were purchased from the Jackson Laboratory (B6C3-Tg(APP695)3Dbo Tg(PSEN1)5Dbo/J; #003378) [27], and then backcrossed to C57BL/6J mice for more than 10 generations after purchase (the resultant strain is designated here as APP/PS1 Tg) [28].

### Cells

Primary cultures were prepared from B6 mice or IL-19^-/-^ mice. Primary microglia cultures were isolated from primary mixed glial-cell cultures prepared from newborn mice using the “shaking off” method (at 14 days *in vitro*), as described previously [29]. The purity of the cultures was > 99%, as determined by anti-CD11b immunostaining. Cultures were maintained in Dulbecco’s modified Eagle’s minimum essential medium (DMEM) (Sigma-Aldrich) supplemented with 10% fetal bovine serum (FBS) (Equitech-Bio, Inc., Kerrville, TX, USA), 5 μg/ml bovine insulin (Sigma-Aldrich), and 0.2% glucose. Primary astrocyte cultures were purified from primary mixed glial cultures by three to four repetitions of trypsinization and replating, as described previously [30]. The purity of astrocytes was > 95%, as determined by GFAP-specific immunostaining. Primary neuron cultures were prepared from the cortices of mouse embryos at embryonic day 17 (E17), as described previously [31]. Briefly, cortical fragments were dissociated into single cells in dissociation solution (Sumitomo Bakelite, Akita, Japan) and resuspended in nerve culture medium (Sumitomo Bakelite). Neurons were plated on polyethylenimine-coated glass coverslips (Asahi Techno Glass, Chiba, Japan) at a density of 5 × 10^4^ cells/well (500 μl/well) in 24-well multidishes. The purity of the cultures was > 95%, as determined by NeuN-specific immunostaining. Neurons were used at 14 days *in vitro* for the following assessments.

### RNA extraction, cDNA microarrays, and reverse transcription-polymerase chain reaction (RT-PCR)

Microglia and astrocytes were seeded at a density of 1 × 10^5^ cells/well (500 μl/well) in 48-well plates. Neurons were plated on polyethylenimine-coated coverslip at a density of 5 × 10^4^ cells/well (500 μl/well) in 24-well plates. Microglia were treated with 100 ng/ml LPS for 0–24 h or 1–1000 ng/ml for 6 h. Astrocytes were treated with 100 ng/ml LPS for 0–24 h or 1–1000 ng/ml for 12 h. Total RNA was extracted from microglia, astrocytes, neurons, hippocampi of APP/PS1 Tg mice and B6 mice (10- and 15-month-old) using the miRNeasy Mini Kit (Qiagen, Valencia, CA, USA). RNA was run on an Agilent 2100 Bioanalyzer to assess quality; only samples with excellent RNA quality (RNA Integrity Number [RIN] > 7) were used. For microarray experiments, RNA samples were evaluated on the Agilent Mouse SurePrint G3 8 × 60K Microarray. The resultant signals were quantitated and analyzed using the Agilent GeneSpring 12.1 software. For RT-PCR experiments, RNA was reverse transcribed into cDNA using SuperScript III (Life Technologies, Carlsbad, CA, USA). Expression levels of genes encoding IL-19, IL-6, TNF-α, hypoxanthine phosphoribosyltransferase 1 (HPRT1), and glyceraldehyde 3-phosphate dehydrogenase (GAPDH) were measured using quantitative PCR (qPCR), which was performed on cDNA on a Rotor-Gene Q using the Rotor-Gene SYBR Green PCR Kit (Qiagen). Relative expression levels were determined using the ΔΔC_T_ method; the genes of interest were normalized to the geometric mean of HPRT1 and GAPDH. The following specific primer sets were used:

Mouse IL-1 sense: 5’-TACAGAGACAGGGTGTTCCAGGAC-3’;

Mouse IL-19 antisense: 5’-GCATTGGTGGCTTCCTGACTGCAGT-3’;

Mouse IL-6 sense: 5’-ACAAGTCGGAGGCTTAATTACACAT-3’;

Mouse IL-6 antisense: AATCAGAATTGCCATTGCACAA-3’;

Mouse TNF-α sense: GACCCTCACACTCAGATCATCTTCT-3’;

Mouse TNF-α antisense: 5’-CCACTTGGTGGTTTGCTACGA-3’;

Mouse HPRT1 sense: 5’-CCTAAGATGAGCGCAAGTTGAA-3’;

Mouse HPRT1 antisense: 5’-CCACAGGACTAGAACACCTGCTAA-3’

Mouse GAPDH sense: 5′-TGTGTCCGTCGTGGATCTGA-3’

Mouse GAPDH antisense: 5′- CCTGCTTCACCACCTTCTTGA-3’

Assays were carried out in five independent trials.

### Enzyme-linked immunosorbent assay (ELISA)

Microglia and astrocytes were seeded at a density of 1 × 10^5^ cells/well in 48-well plates, and then treated with 100 ng/ml LPS for 0–24 h. Hippocampi of APP/PS1 Tg and B6 mice (15-month-old) were collected after transcardial perfusion with phosphate-buffered saline. Concentrations of IL-19, IL-6, and TNF-α in culture supernatant were determined using the appropriate ELISA kits (IL-19, eBioscience; IL-6 and TNF-α, R&D Systems). Assays were carried out in three independent trials.

### Western blotting

Levels of IL-20Rα, IL-20Rβ, STAT3, and phosphorylated STAT3 (pSTAT3) were evaluated by Western blotting as described previously [32]. To evaluate the expression levels of IL-20Rα and IL-20Rβ, microglia and astrocytes were seeded at a density of 1 × 10^5^ cells/well (500 μl/well) in 48-well plates and treated with or without 100 ng/ml LPS for 24 h. To assess the phosphorylation of STAT3, cells were seeded at a density of 1 × 10^5^ cells/well in 48-well plates and treated with 100 ng/ml IL-19 for 0–120 min. Cells were lysed with TNES buffer (50 mM Tris-HCl [pH 7.5], 150 mM NaCl, 1% Nonident P-40, 2 mM EDTA, 0.1% SDS) containing phosphatase inhibitor cocktail (Sigma-Aldrich) and protease inhibitor mixture (Roche, Mannheim, Germany). Fifty micrograms of cell lysate protein dissolved in Laemmli sample buffer were separated on 4–20% SDS-polyacrylamide gels (Mini-Protean TGX, Bio-Rad, Hercules, CA, USA), and transferred to Hybond-P polyvinylidene difluoride membranes (GE Healthcare, Buckingham, UK). The membranes were blocked with 5% skim milk in Tris-buffered saline containing 0.05% Tween-20 for 1 h at room temperature, and then incubated overnight at 4°C with rabbit anti-IL-20Rα polyclonal antibodies (Merck Millipore, Billerica, MA, USA), rat anti-IL-20Rβ monoclonal antibody (eBioscience, San Diego, CA, USA), mouse anti-STAT3 monoclonal antibody (BD Pharmingen, Franklin Lakes, NJ, USA), rabbit anti-pSTAT3 polyclonal antibodies (Cell Signaling Technology, Danvers, MA, USA), or mouse anti-β-actin monoclonal antibody (Sigma-Aldrich), followed by incubation with horseradish peroxidase—conjugated secondary antibodies (GE Healthcare, Buckingham, UK) for 1 h at room temperature. The signals were visualized using SuperSignal West Pico chemiluminescent substrate (Thermo Fisher Scientific), and quantitated using a CS Analyzer 3.0 system (Atto, Tokyo, Japan). Assays were carried out in five independent trials.

### Cell proliferation assay

Microglia were treated with IL-19 (0–100 ng/ml) for 24 h. Cell proliferation was assessed using the 3-(4,5-dimethyl-thiazol-2yl)-5-(3-carboxymethoxyphenyl)-2-(4-sulfophenyl)-2H-tetrazolium (MTS) assay with the CellTiter 96 AQueous One Solution assay (Promega, Madison, WI, USA) as described previously [33]. Assays were carried out in five independent trials.

### Statistical analysis

Statistical significance was analyzed using Student’s *t*-test or one-way analysis of variance (ANOVA) followed by post-hoc Tukey’s test, using GraphPad Prism version 6.0 (GraphPad Software, La Jolla, CA, USA).

## Results

### Activated microglia are the predominant producers of IL-19

First, we evaluated the mRNA expression profile of activated microglia using cDNA microarrays. We found that 1,449 genes were significantly upregulated following treatment with LPS for 24 h. We identified IL-19 as the most upregulated gene (Table 1).

**Table 1:** Upregulated genes in activated microglia (Top 10).

Gene name	Fold Change (vs. untreated)
interleukin-19	7287.7
schlafen 4	5064.0
cDNA sequence U90926	4117.9
urate (5-hydroxyiso-)hydrolase	2273.6
interleukin-6	1852.3
solute carrier organic anion transporter family, member 2b1	1502.6
nitric oxide synthase 2, inducible	1022.8
interleukin-1 family, member 6	980.2
prostaglandin E synthase	915.8
immunoresponsive gene 1	908.5

Gene expression levels are expressed as fold change relative to the untreated control (n = 3).

**Fig 1:**
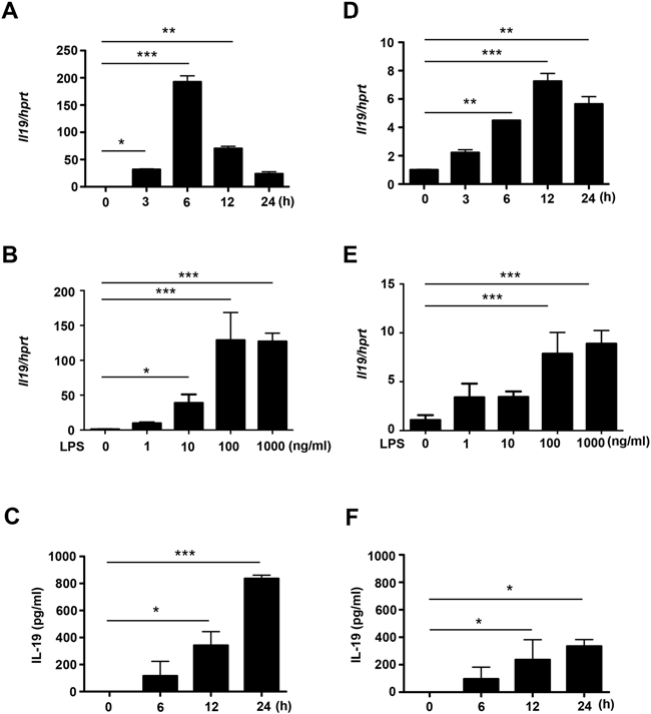
Activated microglia are the predominant source of IL-19 in the CNS. (A) qPCR data for IL-19 in microglia treated with LPS (100 ng/ml) for 0–24 h. (B) qPCR data for IL-19 in microglia treated with LPS (0–1000 ng/ml) for 6 h. (C) ELISA data for IL-19 in culture supernatant of microglia treated with LPS (100 ng/ml) for 24 h. (D) qPCR data for IL-19 in astrocytes treated with LPS (100 ng/ml) for 0–24 h. (E) qPCR data for IL-19 in astrocytes treated with LPS (0–1000 ng/ml) for 12 h. (F) ELISA data for IL-19 in culture supernatant of astrocytes treated with LPS (100 ng/ml) for 24 h. Microglia released approximately twice as much IL-19 as astrocytes. *, *p* < 0.05. **, *p* < 0.01. ***, *p* < 0.001. Values are means ± SD (n = 5).

Next, we confirmed IL-19 expression in CNS cells using qPCR and ELISA. LPS stimulation significantly induced IL-19 mRNA and protein expression in microglia and astrocytes (Fig. 1), but not in neurons (data not shown). IL-19 mRNA expression levels in microglia and astrocytes reached their peaks at 6 h and 12 h after stimulation with 100 ng/ml LPS, respectively (Fig. 1, A and D). Next, we evaluated IL-19 mRNA expression levels in microglia and astrocytes stimulated with LPS at graded doses (0–1000 ng/ml) for 6 h and 12 h, respectively. IL-19 mRNA reached a plateau at 100 ng/ml LPS (Fig. 1, B and E). Production of IL-19 in microglia and astrocytes increased in a time-dependent manner, and microglia produced approximately twice as much IL-19 as astrocytes (Fig. 1, C and F). These results indicate that IL-19 is predominantly produced by activated microglia in the CNS.

### Functional IL-19 receptor is exclusively expressed in microglia

**Fig 2:**
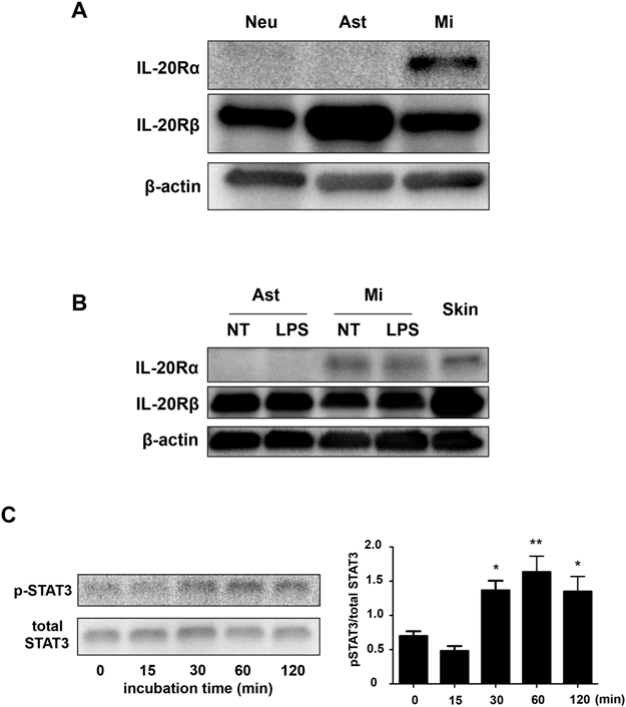
Microglia are the only cells in the CNS that express functional IL-19 receptor. (A) Western blotting data for receptors of IL-19. Only microglia express both the IL-20Rα and IL-20Rβ subunits. Neu, neurons; Ast, astrocytes; Mi, microglia. (B) Western blotting data for receptors of IL-19. LPS stimulation did not change the expression levels of IL-19 receptor subunits. Skin was used as the positive control. NT, untreated; LPS, LPS-treated. (C) Western-blotting analysis of STAT3 phosphorylation in microglia treated with 100 ng/ml IL-19. IL-19 induced STAT3 phosphorylation, the main downstream signal of the IL-19 receptor. All quantitative data are expressed as means ± SD (n = 3). *, *p* < 0.01 *vs*. 0 min. **, *p* < 0.001 *vs*. 0 min.

**Fig 3:**
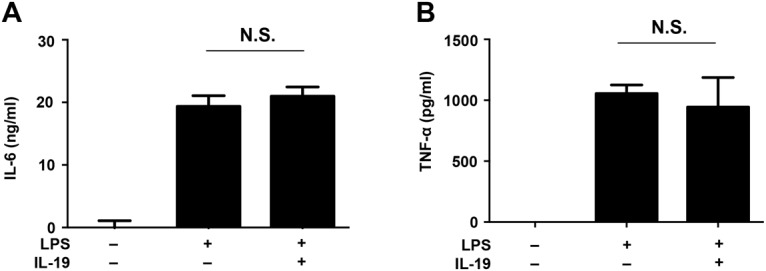
IL-19 does not suppress production of pro-inflammatory cytokines in LPS-stimulated astrocytes. Astrocytes were treated with LPS (100 ng/ml) and recombinant IL-19 (100 ng/ml) for 24 h. (A) Production of IL-6 and (B) TNF-α. Treatment with IL-19 did not microglial production of IL-6 and TNF-α. Values are means ± SD (n = 3). N.S., not significant.

To determine which cells can respond to IL-19 in the CNS, we examined the levels of the IL-19 receptor (a heterodimer of the IL-20Rα and IL-20Rβ subunits) in CNS cells by Western blotting. A recent study reported that IL-19 receptor is expressed in astrocytes, but not in microglia [34]. However, our data indicated that the IL-20Rα subunit was exclusively expressed in microglia, whereas the IL-20Rβ subunit was ubiquitously expressed in neurons, astrocytes, and microglia (Fig. 2A). Moreover, LPS stimulation did not alter the expression levels of the IL-19 receptor subunits in astrocytes and microglia (Fig. 2B). Downstream signals of IL-19 reportedly mediate STAT3 phosphorylation [20,21,35]. Indeed, Western-blotting analysis confirmed that treatment with IL-19 induced STAT3 phosphorylation in microglia (Fig. 2C). Although a previous study also showed that IL-19 suppresses the production of IL-6 and TNF-α in LPS-stimulated astrocytes [34], in our hands IL-19 did not affect the levels of production of these cytokines by LPS-stimulated astrocytes (Fig. 3, A and B). These data show that microglia exclusively express the functional receptor of IL-19, and are thus the primary potential targets of IL-19 in the CNS.

### IL-19 does not activate microglia in resting state

**Fig 4:**
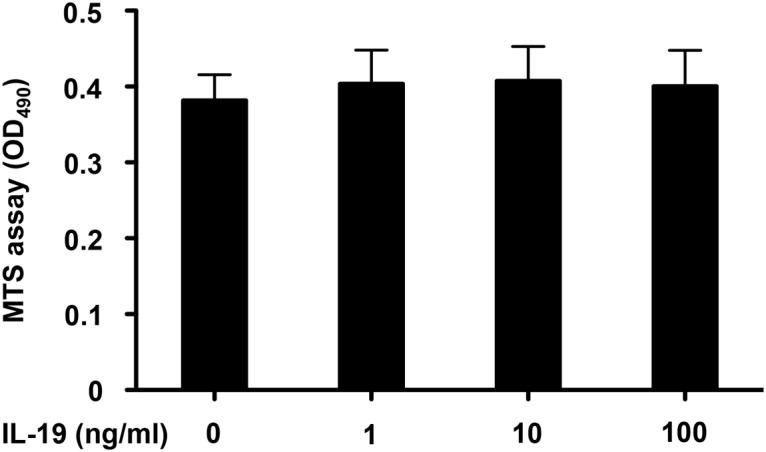
IL-19 does not affect microglial proliferation. Microglial proliferation was evaluated by MTS assay. Treatment with 1–100 ng/ml IL-19 for 24 h did not affect microglial proliferation. Values are means ± SD (n = 5).

**Fig 5:**
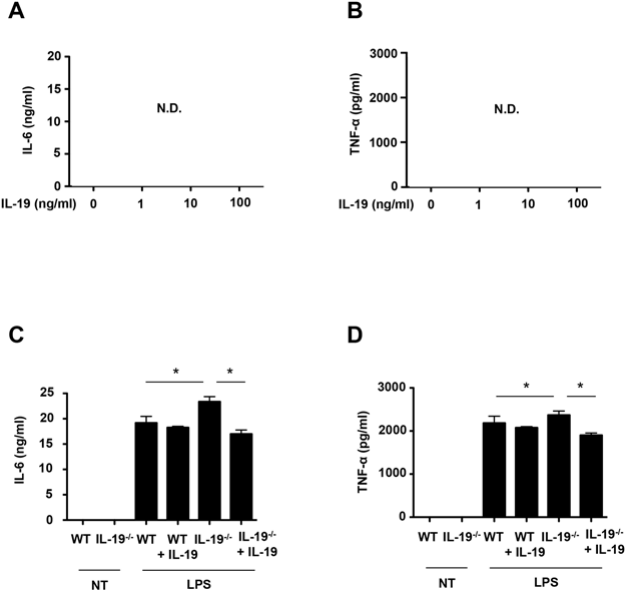
IL-19 suppresses production of pro-inflammatory cytokines in activated microglia. (A and B) Microglia were treated with recombinant IL-19 (100 ng/ml) for 24 h. Production of (A) IL-6 and (B) TNF-α. Stimulation with IL-19 itself did not induce microglial production of IL-6 and TNF-α. (C and D) Microglia from B6 mice and IL-19^-/-^ mice were incubated in the presence of 100 ng/ml LPS, with or without 100 ng/ml IL-19, for 24 h. Production of (C) IL-6 and (D) TNF-α. IL-19 deficiency increased the production of pro-inflammatory factors in activated microglia, and this effect was reversed by addition of IL-19. *, *p* < 0.001. Data indicate means ± SD (n = 3). N.D., not detected.

Next, we investigated whether IL-19 affects the proliferation of microglia. The MTS assay revealed that stimulation with IL-19 itself did not affect microglial proliferation (Fig. 4). Similarly, treatment with IL-19 did not induce microglial production of pro-inflammatory cytokines such as IL-6 and TNF-α (Fig. 5, A and B).

### IL-19 suppresses the production of pro-inflammatory factors in activated microglia

Next, we investigated whether IL-19 affects the functions of activated microglia. Additional treatment of IL-19 did not significantly suppress the production of IL-6 and TNF-α in LPS-stimulated microglia, but ablation of IL-19 increased the production of these cytokines in LPS-stimulated microglia (Fig. 5, C and D), and these effects were reversed by addition of IL-19 (Fig. 5, C and D). These results indicate that IL-19 may serve to limit microglial activation by suppressing pro-inflammatory cytokine production.

### In APP/PS1 Tg mice, IL-19 is upregulated in affected areas in association with disease progression

**Fig 6:**
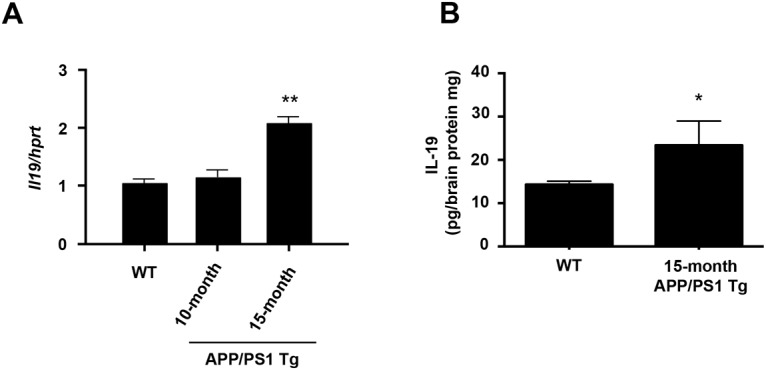
In a mouse model of AD, IL-19 is upregulated in affected regions in association with disease progression. (A) qPCR data for IL-19 in the hippocampi of APP/PS1 Tg and B6 mice. (B) ELISA data for IL-19 in the hippocampi of APP/PS1 Tg and B6 mice. IL-19 expression level gradually increased as disease progressed. WT, wild-type B6 mice. *, *p* < 0.01 *vs*. WT. **, *p* < 0.001 *vs*. WT. Values are means ± SD (n = 3).

Finally, to assess the involvement of IL-19 in neurodegenerative diseases, we investigated the expression pattern of IL-19 in a mouse model of AD using APP/PS1 Tg mice. mRNA expression levels of IL-19 gradually increased in the hippocampi of APP/PS1 Tg mice (Fig. 6A). Protein levels of IL-19 were also significantly elevated in the diseased mice (Fig. 6B).

## Discussion

In this study, we showed that activated microglia secrete IL-19 and are the only CNS cells to express functional IL-19 receptor. Although a recent paper [34] reported that astrocytes express IL-19 and its receptor, our findings revealed that microglia are both the main source and target of IL-19 in the CNS. Previous studies reported that IL-19 is derived primarily from macrophages, and to a lesser extent from B cells and epithelial-lineage cells including epithelial cells, endothelial cells, and keratinocytes [36–38]. Our data also demonstrated that microglia, the macrophage-like cell in the CNS, produce more IL-19 than astrocytes, which belong to the epithelial lineage.

Previous reports showed that monocyte-lineage cells release IL-19 upon pro-inflammatory stimulation by factors such as LPS, IL-6, IL-17, and TNF-α [39,40]. IL-19 induces monocytes to produce IL-6 and TNF-α [22,39] and triggers T cells to release T-helper type 2 cytokines including IL-4, IL-5, IL-10, and IL-13 [23]. Therefore, IL-19 is recognized as an important player in the pathogenesis of systemic inflammatory diseases such as psoriasis, asthma, rheumatoid arthritis, and sepsis [22–24,41]. On the other hand, IL-19 also exerts anti-inflammatory activities in inflammatory bowel disease and vascular inflammatory diseases [25,42]. In contrast to its pro-inflammatory properties in monocytes, IL-19 attenuates the production of IL-6 and TNF-α in macrophages [25]. Our data also demonstrated that IL-19 has an anti-inflammatory activity in microglia. Activation of STAT3 is a key downstream step in IL-19 receptor signaling [20,21,35], and our data also showed that stimulation with IL-19 activates STAT3 in microglia, as shown previously in macrophages [25]. Moreover, IL-19 reportedly suppresses inflammatory responses in vascular smooth muscle cells via downregulation of human antigen R, which promotes transcription of pro-inflammatory factors [42]. Thus, the pleiotropic properties of IL-19 in monocyte-lineage cells may depend on signaling downstream of the IL-19 receptor.

Activated microglia play a pivotal role in the pathogenesis and progression of various neurological disorders such as trauma, stroke, inflammation, epilepsy, and neurodegenerative diseases by releasing neurotoxic factors including glutamate, nitric oxide, and reactive oxygen species [6–12,43]. Microglia also exacerbate neuroinflammation associated with leukocyte infiltration by secreting pro-inflammatory cytokines and chemokines including TNF-α, interferon-γ (IFN-γ), IL-1β, IL-6, IL-12, IL-17, and IL-27 [5,43–47]. These microglial inflammatory and neurotoxic factors fuel the spiral of microglial activation and sustain chronic neuroinflammation and neurodegeneration [1,5,43,48]. On the other hand, activated microglia also utilize self-limiting systems. IFN-γ, a well-known microglia stimulatory factor, is released from activated microglia [44,47] but it also alleviates microglia-mediated neuroinflammation in manner that depends on activation-induced cell death [49], providing a possible mechanism for relapse and remission in multiple sclerosis. Our data suggest that IL-19 may participate in a novel mechanism by which microglia self-limit their inflammatory response. Unlike IFN-γ, IL-19 did not affect microglial survival. Furthermore, supplemental IL-19 did not exert additional suppressive effects on pro-inflammatory cytokine production by activated microglia, probably because these cells had already released a saturating amount of IL-19. Therefore, IL-19 may be a weaker negative regulator of microglia than IL-10. Mouse model of AD exhibited gradual upregulation of IL-19 in the affected areas as disease progressed. Further studies are needed to clarify whether additional administration of IL-19 could effectively slow and halt the progression of neurodegenerative diseases.

In summary, we identified IL-19 as a novel anti-inflammatory cytokine released from activated microglia. In the CNS, microglia are the only cells that express functional IL-19 receptor; therefore, microglia are both the main source and target of IL-19. Ablation of IL-19 increased microglial production of pro-inflammatory factors, and this effect was reversed by addition of IL-19. Our findings suggest that microglia self-limit their participation in neuroinflammation and neurodegeneration by producing a negative regulator, IL-19.
